# Bone targeted treatments in cancer – The story so far

**DOI:** 10.1016/j.jbo.2016.03.002

**Published:** 2016-04-23

**Authors:** Robert Coleman

**Affiliations:** University of Sheffield Weston Park Hospital, Sheffield S10 2SJ, United Kingdom

**Keywords:** Bisphosphonates, Cancer, Metastasis, Bone loss

Bone targeted treatments have transformed the quality of life and physical functioning of patients with metastatic bone disease and, in certain circumstances, have been shown to prevent the development of bone metastases and improve survival. However, the steps in development of these agents have been somewhat disjointed and based on good fortune as well as scientific endeavour and rational clinical development.

Although bisphosphonates were first synthesised in the late 19th century, initial usage was restricted to a range of industrial processes and their potential clinical relevance was not appreciated until the late 1960s [1]. Then, following development for the treatment of osteoporosis and Paget's disease of bone, a few academic groups began to investigate the role of bisphosphonates in oncology, primarily with a focus on hypercalcaemia of malignancy, which 25 years ago was a common metabolic, life threatening complication of advanced cancer. The treatments at the time such as calcitonin, mithramycin and corticosteroids in addition to intravenous rehydration were all sub-optimal and typically only controlled serum calcium for a few days so there was a major unmet clinical need for a safe and effective treatment strategy.

The early trials focused on the use of etidronate and clodronate with some success [2]. Initially, there were concerns that intravenous aminobisphosphonates, such as pamidronate, would result in renal damage and for this reason, very low dose administration (1 mg daily) was first used [3]. Subsequently, larger single doses were shown to be safe and effective [4], [5], ultimately leading to the regulatory approval of pamidronate 60–90 mg as a single infusion alongside intravenous fluids for hypercalcaemia of malignancy.

In the 1980s, small trials with oral clodronate and oral pamidronate had suggested useful effects on skeletal morbidity in breast cancer and multiple myeloma [6], [7]. Subsequently, a number of European investigators showed that intermittent intravenous pamidronate had clinically useful effects on bone pain from metastatic disease and induced radiographic sclerosis of lytic metastatic lesions [8], [9]. The latter imaging change was initially interpreted as a possible antitumour effect with healing of lytic metastases. However, this was almost certainly not the case but simply reflected the effects of treatment uncoupling bone resorption and formation with specific inhibition of tumour induced osteolysis but continued new bone formation. However, as a result of these pilot studies, Ciba Geigy, the manufacturer of pamidronate at the time, initiated large randomised clinical trials to evaluate the effects of intravenous pamidronate every 3–4 weeks on disease progression and a new endpoint termed skeletal related events (SREs) to objectively reflect the skeletal morbidity associated with metastatic bone disease. SREs included radiotherapy to bone for pain relief or structural damage, pathological fracture, spinal cord compression, orthopaedic intervention for impending or actual fracture and hypercalcaemia. These studies [10], [11], [12], and similar trials with oral clodronate [13], showed significant benefits in breast cancer and multiple myeloma patients with a 25–30% reduction in SREs, reduced pain and improved quality of life. As a result of these trials intravenous pamidronate or, in some health care settings, oral clodronate became part of standard management for patients with bone involvement from breast cancer or multiple myeloma since the mid 1990s.

In the late 1990s, more potent bisphosphonates were developed; both oral ibandronate and intravenous zoledronic acid were evaluated with the latter showing slight superiority over pamidronate for breast cancer patients [14]. Additionally, placebo controlled trials in castrate resistant prostate cancer [15] and other solid tumours [16] associated with bone metastases (e.g. lung and renal cancers) showed that SREs were significantly reduced with zoledronic acid and this agent became the standard of care throughout most of the world for patients with bone metastases from solid cancers and applied to patients with osteoblastic as well as osteolytic bone metastases. Furthermore, survival in multiple myeloma was improved with zoledronic acid when compared to oral clodronate [17] and thus zoledronic acid became the preferred bisphosphonate also for patients with multiple myeloma.

There have been great strides in our understanding of the biology of bone metastases and the regulation of bone cell function. This included the discovery of RANK ligand [18] and the subsequent development of denosumab, a specific humanised antibody to RANK ligand. Large phase III trials showed that denosumab was somewhat more effective in preventing skeletal morbidity in solid tumours (although not to date in multiple myeloma), was easier to administer as a subcutaneous injection, was associated with fewer renal adverse events and did not induce the acute phase response associated with intravenous aminobisphosphonates [19], [20], [21]. As a result, clinical practice guidelines generally recommend denosumab as the preferred agent [22] although they recognise, that from a health economic perspective now that bisphosphonates are generic, that health care systems may find it difficult from a financial perspective to support denosumab for all clinical situations and zoledronic acid remains a reasonable alternative.

For several decades there has been interest in the potential of bone-targeted agents, through their profound effects on bone physiology, to modify the process of metastasis and have effects on important disease outcomes such as recurrence and survival [23]. This hypothesis has been the focus of extensive laboratory and clinical research in a several tumour types. Multiple clinical trials with bisphosphonates have been performed; no convincing efficacy has been seen in patients with prostate [24] or lung cancers [25] and variable outcomes in terms of disease recurrence reported in breast cancer [26], [27], [28], [29], [30]. We showed that disease outcomes in breast cancer, both in patients [30] and animal models of metastasis [31], were influenced by levels of reproductive hormones and that this appeared to explain the variable results seen in individual clinical trials.

In a recent initiative with the Early Breast Cancer Clinical Trials Group (EBCTCG), individual patient data from around the world were collected to enable a meta-analysis of 18,766 women included in randomised trials that evaluated the effect of an adjuvant bisphosphonates on breast cancer outcomes [32]. With 3453 and 2106 breast cancer recurrences and deaths respectively, this meta-analysis allowed the identification of important clinical benefits that could not be reliably demonstrated in individual trials. Bisphosphonates were shown to reduce first distant recurrence in bone (RR=0.83; 95%CI 0.73–0.94, 2p=0.004) and the meta-analyses confirmed a significant interaction between treatment efficacy and menopausal status. There were no demonstrable benefits in premenopausal women but in 11,767 postmenopausal women, highly significant reductions in bone recurrence (RR=0.72; 95%CI 0.60–0.86, 2p=0.0002) and breast cancer mortality (0.82; 95%CI 0.73–0.93, 2p=0.002) were seen. These findings are currently changing clinical practice and in many countries becoming part of routine adjuvant therapy [22], [33].

Denosumab also has some beneficial effects on the underlying disease with evidence of modest activity in delaying the development of bone metastases in prostate cancer patients with rising PSA levels despite androgen deprivation therapy (ADT) [34]. Preliminary data also suggest possible efficacy in postmenopausal breast cancer patients but definitive results are awaited from the ongoing large phase III D-CARE trial [35].

Finally, the knowledge obtained from studies of bone-targeted treatments in osteoporosis has been applied to the cancer setting. Many patients, notably breast cancer patients receiving aromatase inhibitors or experiencing chemotherapy induced premature menopause and men with prostate cancer receiving ADT experience rapid bone loss due to the resultant suppression of circulating oestradiol levels and are at increased risk of fragility fractures. Both bisphosphonates [36] and denosumab [37] have been shown to prevent this treatment induced bone loss with the effects of denosumab on fracture incidence particularly impressive [37], [38].

In the future we can expect to see the currently available bone-targeted agents applied increasingly widely across multiple disease settings with further gains in quality of life and, potentially in some disease settings, improvements in disease outcomes with fewer bone recurrences and improved patient survival. Additionally, new bone-targeted agents such as radium-223 are already being incorporated into the management of advanced prostate cancer [39] and may have a future role in other diseases [40]. The search is now on for a safe and effective anabolic agent [41] that can augment the efficacy of the many resorption inhibitors we already have available and further improve bone structure and the response to treatments that directly target the cancer.
